# Characterization of Rotavirus RNAs That Activate Innate Immune Signaling through the RIG-I-Like Receptors

**DOI:** 10.1371/journal.pone.0069825

**Published:** 2013-07-23

**Authors:** Dina Uzri, Harry B. Greenberg

**Affiliations:** 1 Departments of Medicine and Microbiology & Immunology, Stanford University School of Medicine, Stanford, California, United States of America; 2 VA Palo Alto Health Care System, Palo Alto, California, United States of America; Virginia Polytechnic Institute and State University, United States of America

## Abstract

In mammalian cells, the first line of defense against viral pathogens is the innate immune response, which is characterized by induction of type I interferons (IFN) and other pro-inflammatory cytokines that establish an antiviral milieu both in infected cells and in neighboring uninfected cells. Rotavirus, a double-stranded RNA virus of the *Reoviridae* family, is the primary etiological agent of severe diarrhea in young children worldwide. Previous studies demonstrated that rotavirus replication induces a MAVS-dependent type I IFN response that involves both RIG-I and MDA5, two cytoplasmic viral RNA sensors. This study reports the isolation and characterization of rotavirus RNAs that activate IFN signaling. Using an *in vitro* approach with purified rotavirus double-layer particles, nascent single-stranded RNA (ssRNA) transcripts (termed *in vitro* ssRNA) were found to be potent IFN inducers. In addition, large RNAs isolated from rotavirus-infected cells six hours post-infection (termed *in vivo* 6 hr large RNAs), also activated IFN signaling, whereas a comparable large RNA fraction isolated from cells infected for only one hour lacked this stimulatory activity. Experiments using knockout murine embryonic fibroblasts showed that RIG-I is required for and MDA5 partly contributes to innate immune signaling by both *in vitro* ssRNA and *in vivo* 6 hr large RNAs. Enzymatic studies demonstrated that *in vitro* ssRNA and *in vivo* 6 hr large RNA samples contain uncapped RNAs with exposed 5’ phosphate groups. RNAs lacking 2’-O-methylated 5’ cap structures were also detected in the *in vivo* 6 hr large RNA sample. Taken together, our data provide strong evidence that the rotavirus VP3 enzyme, which encodes both guanylyltransferase and methyltransferase activities, is not completely efficient at either 5’ capping or 2’-O-methylation of the 5’ cap structures of viral transcripts, and in this way produces RNA patterns that activate innate immune signaling through the RIG-I-like receptors.

## Introduction

Rotavirus (RV) is the primary etiological agent of severe dehydrating diarrhea in young children worldwide, and is responsible for almost half a million deaths annually [1,2]. In 2006, two live attenuated RV vaccines, RotaTeq and Rotarix, were introduced to the market and are now licensed for use worldwide. Studies thus far indicate that these vaccines are both safe and effective at preventing RV-associated disease and mortality although their efficacy is significantly diminished in the poorest countries of Asia and Africa [3].

RV is a member of the *Reoviridae* family of non-enveloped, segmented double-stranded RNA (dsRNA) viruses. The virus exhibits host-range restricted replication, such that viruses from one species (homologous host) are only infrequently isolated from another species (heterologous host) [4,5]. The RV triple layer particle (TLP), encapsidating the eleven dsRNA genome segments, binds to cells via its attachment protein VP4 and enters through the early endosomal pathway. Upon entry, the outer layer of the viral capsid is shed, revealing a transcriptionally active double-layer particle (DLP). The virus-encoded RNA-dependent RNA polymerase, RdRP, uses the minus-strand of the segmented dsRNA genome as a template to generate mRNAs, which are extruded into the cytoplasm. Some of these mRNAs are translated into viral proteins, while others accumulate in cytoplasmic inclusions called viroplasms where they are packaged into newly forming subviral particles. During this encapsidation step, RV plus-strand RNAs undergo a single round of minus-strand replication to generate a complete set of eleven dsRNA genome segments. Capsid formation occurs concurrently with encapsidation and genome replication. Progeny subviral particles bud through the endoplasmic reticulum, where they mature into TLPs that then exit the cell via lysis or exocytosis [6].

Upon infection of mammalian cells with bacterial or viral pathogens, an early line of defense, termed the innate immune response, is triggered. The innate immune response is initiated by host proteins called pattern recognition receptors (PRRs) that sense pathogen-associated molecular patterns (PAMPs) presented during the microbial infection. The innate immune response is characterized by the production of type I interferons (IFN-α/β) and many other pro-inflammatory cytokines [7]. Binding of type I IFNs in an autocrine or paracrine manner to the IFN-α/β receptor initiates a JAK/STAT signaling cascade, which culminates in the upregulation of IFN-stimulated genes (ISGs) [7]. ISGs encode effector proteins such as PKR, OAS, and Mx that create an antiviral environment within infected and neighboring uninfected bystander cells [8]. The Toll-like receptors (TLRs) are the best-characterized family of PRRs. These membrane-bound receptors recognize different microbial signatures either at the cell surface or within the endosomal compartment, and in many cases their expression is limited to innate immune cells such as plasmacytoid dendritic cells (pDCs) and macrophages [9]. More recently, a new family of cytoplasmic PRRs known as the RIG-I-like receptors (RLRs) was identified, and these sensors have been shown to play critical roles in defending against a wide variety of viruses in multiple cell types [10,11]. The RLR family comprises RIG-I, MDA5, and LGP2, and these receptors recognize distinct RNA PAMPs. Thus far, ligands found to activate RIG-I include uncapped 5’-triphosphorylated (5’ ppp) ssRNA [12,13,14,15], 5’ ppp ssRNA containing polyuridine or polyadenine stretches [16,17], 5’ ppp base-paired RNAs [18,19], short dsRNA [20,21], and 3’-monophosphorylated RNA [22]. MDA5 has been shown to detect longer (>2 kb) dsRNA stretches [20] and higher-order RNA structures [23], and has been linked to sensing of RNAs with incompletely 2’-O-methylated 5’ cap structures [24]. RIG-I and MDA5 share similar protein organization, with two tandem N-terminal caspase activation and recruitment domains (CARDs), followed by a DExD/H box helicase domain, and terminating with a regulatory/repressor domain. Unlike RIG-I and MDA5, LGP2 does not contain the N-terminal CARDs, and it has been shown to function either as an activator or repressor of RLR signaling, depending on the virus [25,26,27,28]. Upon binding of RIG-I and MDA5 to their respective RNA ligands, they undergo conformational changes, multimerize, are modified with polyubiquitin chains, and form a CARD-mediated complex with the mitochondrial adaptor protein MAVS (also known as VISA, Cardif, and IPS1) [27,29,30,31,32,33,34]. MAVS serves as a platform for recruitment of kinases and other signaling proteins, leading to activation of the transcription factors IRF3/7 and NF-κB, which are required for induction of type I IFN expression [31,32,33,34].

The adaptive immune response to RV infection has been well characterized, and studies have demonstrated that CD8+ T cells play a critical role in the timely clearance of a primary infection while B cells are crucial for resistance to re-infection [35,36]. Much less is known about the role of innate immune signaling in protection from RV infection, however. Experiments *in vitro* indicate that RV infection induces an early IFN response, but in most cases the virus rapidly suppresses IFN signaling through the actions of viral proteins such as NSP1 [37,38,39,40,41,42,43]. Previous work from our laboratory demonstrated that although RV does not replicate efficiently in human pDCs, it still induces type I IFN production in a process requiring both viral dsRNA and structural proteins [44]. Recently, Sen et al. reported that RV infection of murine embryonic fibroblasts induces a MAVS-dependent early antiviral transcriptional response that involves both RIG-I and MDA5, neither of which was independently essential for IFN production [45]. IFN induction in a RV-infected intestinal epithelial cell line was also shown to require MAVS and involved both RIG-I and MDA5 [46]. These results suggest that RV infection produces both RIG-I and MDA5 PAMPs. Similar conclusions were made in studies with the related reovirus [47]. A previous attempt to identify PAMPs produced by reovirus used genomic dsRNA of different sizes purified from viral particles to demonstrate that the shorter segments preferentially signal through RIG-I and the longer segments preferentially signal through MDA5 [20]. Whether the reovirus dsRNA genome functions as a PAMP during actual infection is not clear however. Despite considerable efforts, the specific RV PAMPs that are detected by and activate the RLRs have, thus far, remained elusive.

In this study, we used a combination of *in vitro* and *in vivo* approaches to isolate and characterize RNAs generated during RV transcription. We demonstrate that RV ssRNA transcripts are a key activator of innate immune signaling. RIG-I is required for and MDA5 contributes to IFN production in response to RV transcripts. Enzymatic characterization studies indicate that RV transcription produces uncapped RNAs with exposed 5’ phosphate groups and RNAs with incompletely 2’-O-methylated 5’ cap structures. These two RNA signatures provide a molecular basis for how RV induces RLR-dependent innate immune signaling.

## Results

### Isolation of immunostimulatory rotavirus RNAs from *in vitro*-transcriptions

In rotavirus (RV) infected cells, plus- and minus-strand RV RNAs are first detectable around 3 hours post-infection (hpi), and the level of transcription increases significantly until 9 to 12 hpi, when the concentration of plus-strand transcripts and dsRNA reaches a maximum [48]. Primary RV transcription takes place in the cytoplasm, where the RIG-I-like receptors (RLRs) reside. Secondary transcription and replication take place in protein-dense viroplasm structures, in which viral RNAs are protected from RNAi degradation [49]. Production of genomic dsRNA takes place in viroplasms concurrently with subviral particle formation, and therefore it is unlikely that naked dsRNA genome segments, either from input or replicating virus, are present in any substantial amount as free molecules in the cytoplasm of infected cells. Based on this knowledge, we hypothesized that RV ssRNA transcripts, and not genomic dsRNA, would likely be the primary immunostimulatory molecules generated during infection. To address this hypothesis, we took two complementary approaches to isolate RV RNAs for subsequent characterization. The first approach was to purify RV RNA products from *in vitro*-transcription (IVT) reactions carried out using highly purified RV double-layer particles (DLPs), and the second approach was to purify RNAs from RV-infected cells.

**Figure 1:**
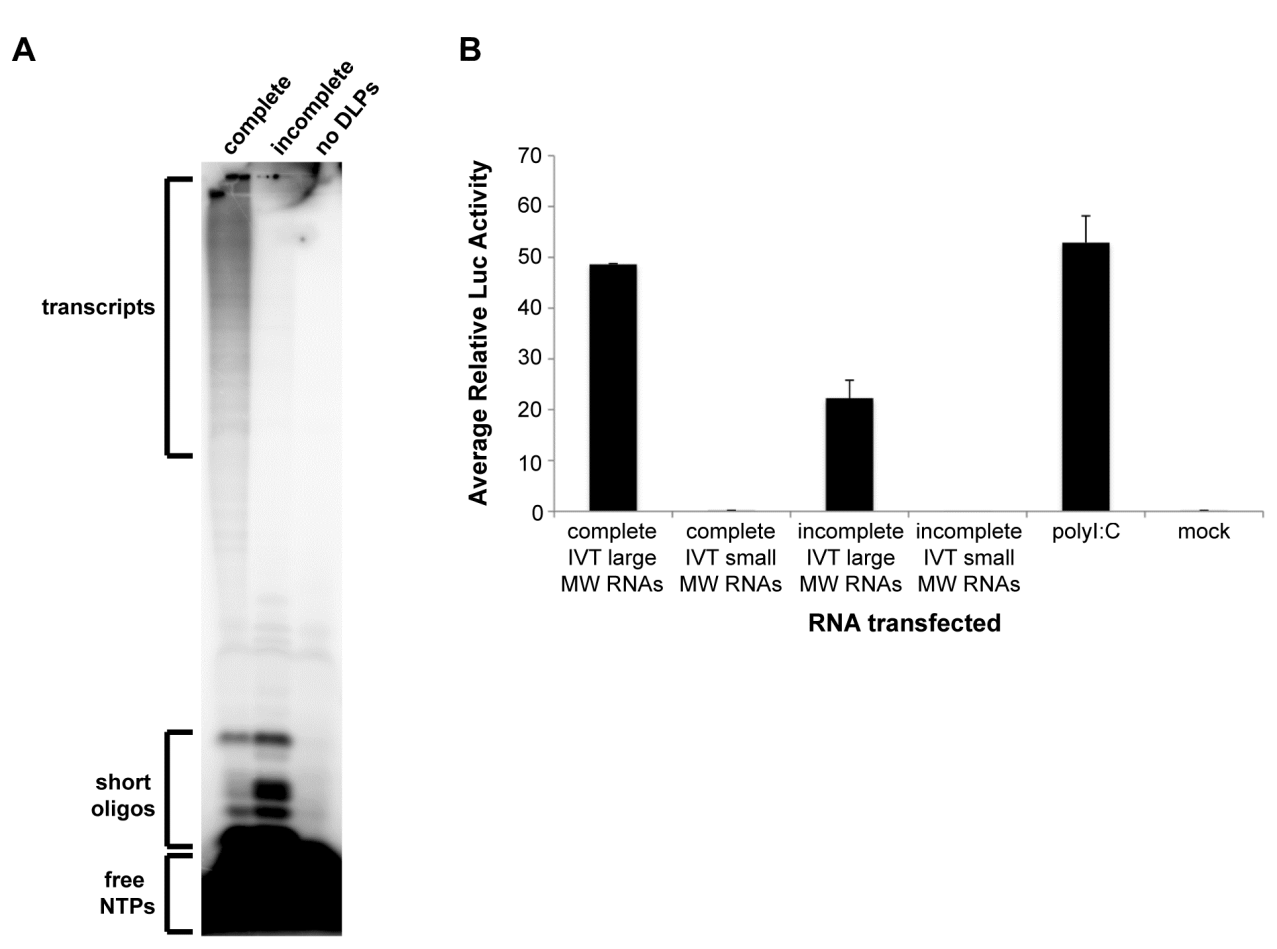
Isolation of immunostimulatory RV RNAs from *in vitro*-transcriptions. (A) *In vitro*-transcription (IVT) reactions were carried out using purified RRV DLPs, α^32^P-GTP, *S*-adenosylmethionine, and all four NTPs (complete), all reaction components except ATP (incomplete), or all reaction components except DLPs (no DLPs). Reactions were carried out as described in the methods and analyzed on a 20% urea-acrylamide gel. (B) Complete and incomplete IVT reactions were carried out and total RNA was purified from the reactions and analyzed on a denaturing urea-acrylamide gel. UV shadowing was used to visualize large and small molecular weight (MW) RNAs, which were then excised from the gel and purified. Huh7 human hepatoma cells were co-transfected with pIFN-beta-luc (firefly luciferase) and pRL-TK (*Renilla* luciferase). After 24 hours (hr), cells were mock-transfected, transfected with the entire recovered large or small MW RNA sample, or transfected with 500 ng/well of polyI:C in duplicate. Approximately 21 hr later, cell lysates were made and analyzed using the dual-luciferase reporter assay system. The firefly luciferase light unit values were divided by the *Renilla* light units (transfection efficiency control) to generate the relative luciferase (luc) activity value. Bars show the average relative luc values plus standard deviation.

DLPs contain the complete dsRNA genome, have a functional RNA-dependent RNA polymerase (RdRP), and can efficiently generate capped and methylated transcripts when supplemented with nucleotides (NTPs), *S*-adenosylmethionine, and magnesium ions [50]. The first question we asked was what types of RNAs are generated in IVT reactions. To address this question, we purified DLPs from MA104 monkey kidney cells infected with the rhesus rotavirus (RRV) strain, and carried out IVT reactions with DLPs, α-^32^P-GTP, and all four NTPs (complete conditions) or in the absence of ATP (incomplete conditions). As a negative control, we included a reaction lacking DLPs. After a 5-hour incubation at 42°C, reactions were treated with antarctic phosphatase to digest unincorporated NTPs into inorganic phosphate (P_i_). RNA products from each reaction were analyzed on a denaturing 20% urea-acrylamide gel. As shown in Figure 1A, nascent transcripts as well as short oligonucleotides (oligos) were generated in the complete IVT reaction. In the incomplete reaction, we detected short oligos but not larger transcripts, and generation of the short oligos was enhanced under incomplete conditions, consistent with several previous reports [51,52]. As expected, no RNA products were detected in the IVT reaction lacking RV DLPs.

To determine if any of the IVT RNA products had immunostimulatory potential, complete and incomplete IVT reactions were carried out under similar conditions but in the absence of α-^32^P-GTP. Total RNA was then purified from the reactions and applied to a denaturing urea-acrylamide gel, and UV shadowing was used to visualize the RNA. In the complete IVT reaction lane, a region of large molecular weight (MW) RNAs was visible beginning at the bottom of the well and extending to approximately 1 centimeter below the well. We did not visualize a region of large MW RNAs in the incomplete IVT reaction lane. The large MW RNAs seen in the complete IVT reaction are presumed to include both nascent transcripts as well as input genomic dsRNA released from the DLPs during the RNA extraction process. However, because the amount of nascent transcripts in the complete IVT sample greatly exceeds that of input genomic dsRNA, we believe that the large MW smear detected by UV shadowing was mostly, if not entirely, due to the nascent transcripts and not the input genomic dsRNA. The incomplete IVT reaction contained an equivalent amount of input genomic dsRNA, but because the actual amount of this dsRNA was low and UV shadowing has limited sensitivity, a large MW RNA smear was not visible in the incomplete IVT reaction lane. A small MW smear at the very bottom of the gel just below the bromophenol blue dye front (expected size of 8 nucleotides) was detected in both IVTs, and this smear likely contained both short oligos and unincorporated NTPs. No RNAs were detected in the middle of the gel in either lane, similar to the findings in Figure 1A. The large MW region at the top of the gel (including the bottom of the well) was excised from the complete IVT lane. A region of comparable size was also excised from the top of the gel in the incomplete IVT lane even though no RNA smear was visible in this region. The small MW smears from the bottom of the gel were excised from both the complete and incomplete IVT lanes. RNAs were eluted from all the gel slices and purified.

To measure the immunostimulatory potentials of these gel-purified RNAs, we transfected the entire recovered large and small MW RNA eluates into Huh7 human hepatoma cells and measured stimulation of an IFN-beta firefly luciferase reporter. The large MW RNA fraction (containing both nascent transcripts as well as input genomic dsRNA) purified from the complete IVT reaction was a potent IFN activator whereas the small MW RNA fraction (containing unincorporated NTPs and short oligos) purified from the complete IVT reaction lacked IFN stimulatory potential (Figure 1B). This result indicates that the large MW RNAs purified from the complete IVT reaction, and not the short oligos, are capable of inducing IFN signaling. Notably, the large MW RNA fraction (containing only input genomic dsRNA) purified from the incomplete IVT reaction was much less stimulatory than the large MW RNA fraction purified from the complete IVT reaction (Figure 1B). This result suggests that although input genomic dsRNA is immunostimulatory, nascent transcripts, which were only generated under the complete reaction conditions, are also capable of eliciting a robust IFN response.

### Lack of detectable dsRNA in and isolation of immunostimulatory RNAs from RV-infected cells

**Figure 2:**
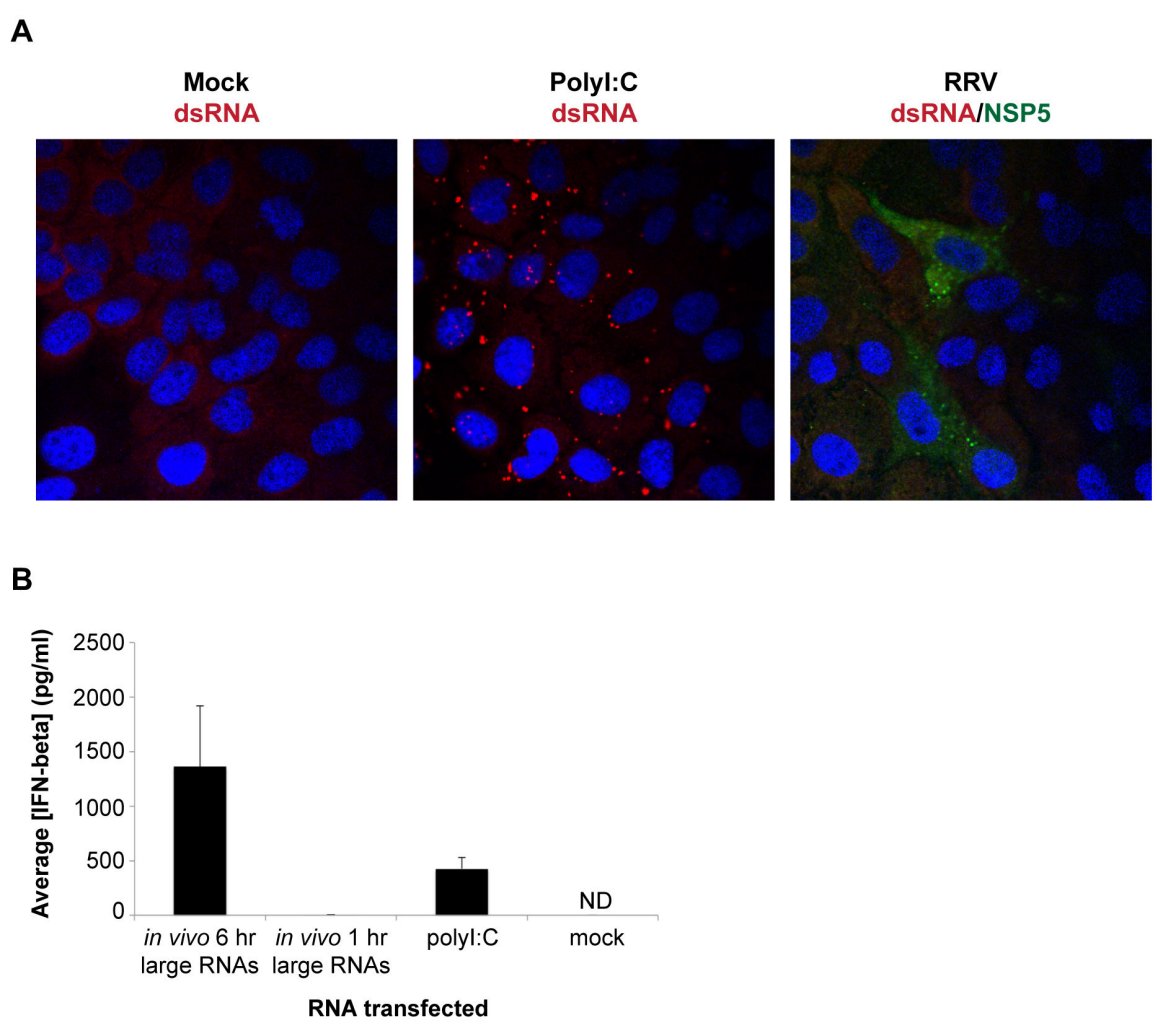
Lack of detectable dsRNA in and isolation of immunostimulatory RNAs from RV-infected cells. (A) MA104 cells in chamber slides were mock-infected, infected with RRV at an estimated MOI of 10, or transfected with 1 μg/well of polyI:C and fixed after a 6-hour (hr) incubation. J2 monoclonal antibody was used to detect dsRNA (red) and NSP5 polyclonal antibody was used to visualize infected cells (green). DAPI was used to stain cell nuclei (blue). (B) Wild type murine embryonic fibroblasts (MEFs) were mock-transfected or transfected with 500 ng/well of *in vivo* 6 hr large RNAs, *in vivo* 1 hr large RNAs, or polyI:C. Approximately 21 hours later, mouse IFN-beta ELISA was used to measure the concentration of secreted IFN-beta protein in the cell media. Bars show the average IFN-beta concentration plus standard deviation. ND, not detected.

In a second approach, we sought to reproduce these results using RNAs generated *in vivo* in RV-infected cells. As an initial step, we wanted to determine whether naked dsRNA can be detected in the cytoplasm of RV-infected cells early in infection, as has been reported previously [53]. MA104 cells were mock-infected, transfected with polyI:C, or infected with RRV at an estimated multiplicity of infection (MOI) of 10. After a 6-hour incubation, cells were fixed and stained with the J2 monoclonal anti-dsRNA antibody, which recognizes dsRNA with a helix size greater than 40 base-pairs (bp) [54]. Punctate dsRNA staining was clearly detectable in the cytoplasm of polyI:C-transfected cells (Figure 2A, middle panel). On the other hand, no RV-associated dsRNA staining was observed at this time-point in RV-infected cells, which were also stained with an antibody to the viral NSP5 protein (Figure 2A, right panel). We also examined infected cells at 8 hours post-infection (hpi), but again did not observe any dsRNA staining (data not shown).

The data from the IVT reactions (Figure 1) and the lack of detectable dsRNA in the cytoplasm of RV-infected cells 6 hpi (Figure 2A) suggested that newly synthesized viral dsRNA was unlikely to be the major immunostimulatory RNA produced during RV infection. To identify other RNA candidates with immunostimulatory potential generated in RV-infected cells, MA104 cells were infected with RRV at an estimated MOI of 5 for 1 or 6 hours (hr), cytoplasmic extracts were generated from the infected cells, and total RNA was isolated from the cytoplasmic extracts. We selected a 6 hr time-point to isolate potentially stimulatory RV RNAs because at 6 hpi primary RV transcription is thought to occur outside of the viroplasm structures [49], and therefore these RV transcripts would be more available for detection by the cytoplasmic RLRs. Total RNA samples isolated from infected MA104 cells were digested with proteinase K and deproteinized by extraction. To selectively remove the excess quantity of small cellular RNAs (i.e. rRNAs and tRNAs) from this total RNA sample, we used 2.5 M lithium chloride (LiCl) to precipitate RNAs over 100 nucleotides (nt), resulting in a large RNA fraction (termed *in vivo* large RNAs). Equivalent amounts of the 1 hr large RNA fraction (containing input RV genomic dsRNA but little if any newly synthesized RV ssRNA transcripts or newly synthesized genomic dsRNA) and 6 hr large RNA fraction (containing input RV genomic dsRNA, newly synthesized RV ssRNA transcripts, and some newly synthesized genomic dsRNA) were transfected into wild type murine embryonic fibroblasts (MEFs). Cell culture media was collected 21 hours post-transfection and the amount of secreted IFN-beta quantified using ELISA. As shown in Figure 2B, the large RNA fraction isolated from MA104 cells infected with RV for 6 hours, but not 1 hour, was highly immunostimulatory. These results indicate that 1) the amount of RV genomic dsRNA present in the input inoculum is not sufficient to induce IFN, and 2) production of immunostimulatory RNAs *in vivo* requires RV transcription and/or replication.

### Role of the RIG-I-like receptors in detection of RV *in vitro* and *in vivo* immunostimulatory RNAs

The next step of our study was to characterize the role of the RIG-I-like receptors (RLRs) in downstream innate immune signaling from the *in vitro*- and *in vivo*-generated RNA samples. For this set of experiments, it was important to use a more homogenous IVT RNA sample as starting material than the gel-purified large MW RNA fraction used previously (Figure 1). We took advantage of a method developed for reverse genetics studies with the related Bluetongue virus [55] to specifically purify RV ssRNA transcripts from the complete IVT reaction. In this method, input DLPs, containing genomic dsRNA, are removed from the IVT reaction by tandem ultracentrifugation followed by selective precipitation of ssRNA from the supernatant using 2 M LiCl. We refer to this sample as *in vitro* ssRNA in the remainder of the paper.

**Figure 3:**
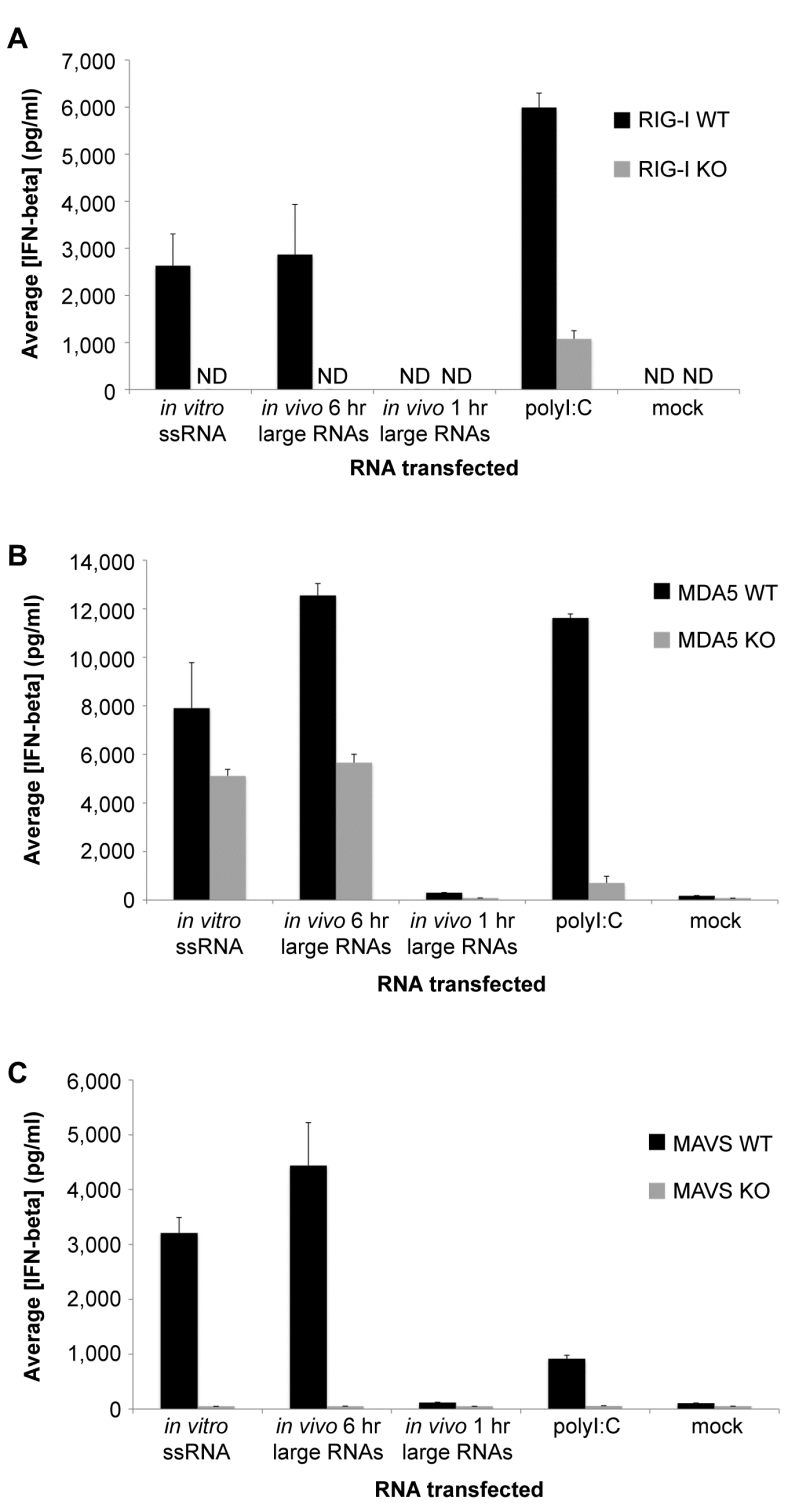
Role of the RLRs in detection of RV *in vitro* and *in vivo* immunostimulatory RNAs. (A) Matched RIG-I wild type (WT) and knock-out (KO) MEFs were mock-transfected or transfected with 500 ng/well of *in vitro* ssRNA, *in vivo* 6 hr large RNAs, *in vivo* 1 hr large RNAs, or polyI:C. (B) Matched MDA5 WT and KO MEFs were transfected exactly as in A. (C) Matched MAVS WT and KO MEFs were transfected exactly as in A. (A–C) Approximately 21 hours later, mouse IFN-beta ELISA was used to measure the concentration of secreted IFN-beta protein in the cell media. Bars show the average IFN-beta concentration plus standard deviation. ND, not detected.

To assess the contribution of the RLRs to sensing of the *in vitro* ssRNA and *in vivo* large RNA samples, matched RIG-I wild type (WT) and knock-out (KO), MDA5 WT and KO, and MAVS WT and KO MEFs were mock-transfected or transfected with equal amounts of *in vitro* ssRNA, *in vivo* 6 hr large RNAs, *in vivo* 1 hr large RNAs, or polyI:C. Cell culture media was collected 21 hours post-transfection and the amount of secreted IFN-beta quantified using ELISA. In all three sets of WT MEFs, the *in vitro* ssRNA and *in vivo* 6 hr large RNA samples were potent inducers of IFN-beta production, whereas the *in vivo* 1 hr large RNA sample was minimally stimulatory (Figure 3A–C). IFN-beta induction in response to the *in vitro* ssRNA and *in vivo* 6 hr large RNA samples was completely abrogated in the absence of RIG-I (Figure 3A), suggesting that these RNA samples require RIG-I for downstream IFN induction. The IFN-beta responses to the *in vitro* ssRNA and *in vivo* 6 hr large RNA samples were only reduced by approximately 50% in the absence of MDA5 (Figure 3B), indicating that these RNAs can be sensed by MDA5, but do not strictly require it for IFN signaling. The mitochondrial adaptor protein MAVS was absolutely necessary for IFN-beta induction in response to both the *in vitro* ssRNA and *in vivo* 6 hr large RNA samples (Figure 3C), consistent with their signaling through the RLRs. IFN-beta induction by polyI:C was diminished to a large extent in the absence of either RIG-I or MDA5 (Figure 3A and B), consistent with its ability to signal through both RLRs.

### Enzymatic characterization of RV *in vitro* and *in vivo* immunostimulatory RNAs

RV RNA synthesis is carried out by the endogenous, virion-associated RdRP, VP1, and the virion-associated guanylyltransferase and methyltransferase, VP3 [6]. RV transcripts possess a type I 5’ cap structure, m^7^GpppGm [56]. We postulated that if capping by VP3 is not 100% efficient, some of the RV mRNAs extruded into the cytoplasm might have exposed 5’ phosphates, a known RIG-I ligand [12,13]. Second, if the VP3 methyltransferase activity is not completely efficient, then some of the transcripts may contain incompletely 2’-O-methylated 5’ caps, a molecular signature that has been shown to induce IFN in an MDA5-dependent manner [24].

**Figure 4:**
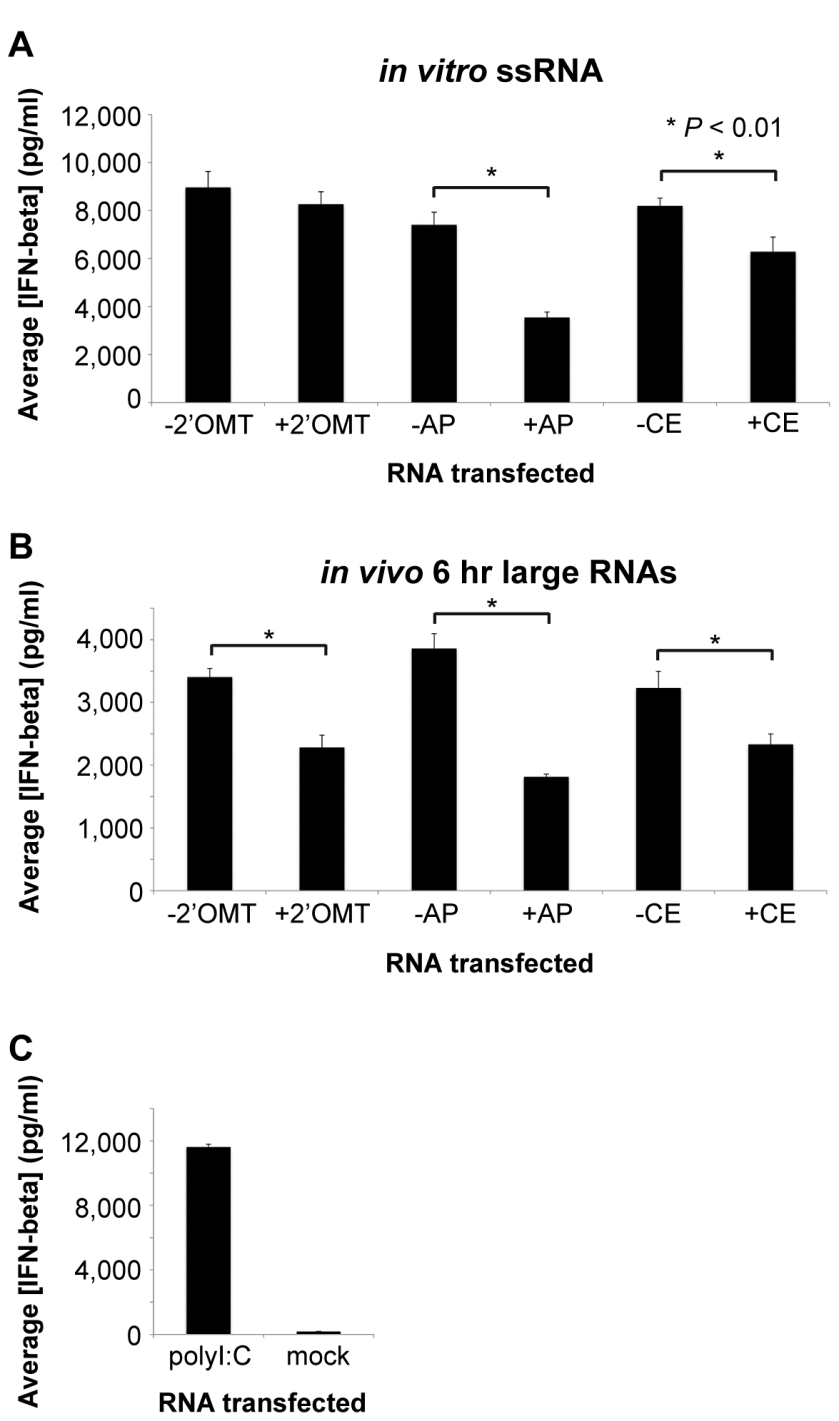
Enzymatic characterization of RV *in vitro* and *in vivo* immunostimulatory RNAs. (A) 2’-O-methyltransferase (2’OMT), antarctic phosphatase (AP), and 5’ capping (CE) reactions were set up using *in vitro* ssRNA. (B) Similar enzymatic reactions were carried out with the *in vivo* 6 hr large RNA sample as in A. (A and B) Treated RNAs were purified and WT MEFs were transfected with 500 ng/well of the indicated RNA sample. (C) WT MEFs were mock-transfected or transfected in triplicate with 500 ng/well of polyI:C as negative and positive controls, respectively. (A–C) Approximately 21 hours later, mouse IFN-beta ELISA was used to measure the concentration of secreted IFN-beta protein in the cell media. Bars show the average IFN-beta concentration plus standard deviation. Data shown are from one representative experiment out of three. * *P* < 0.01 (unpaired Student’s *t*-test).

To elucidate the molecular nature of the RV PAMP(s) found within the *in vitro* ssRNA and *in vivo* 6 hr large RNA samples, we performed a set of enzymatic assays to detect the presence of exposed 5’ phosphate groups or incompletely 2’-O-methylated 5’ cap structures. We incubated the *in vitro* ssRNA and *in vivo* 6 hr large RNA samples with: 2’-O-methyltransferase to add a 2’-O-methyl group to any transcripts that were incompletely methylated, antarctic phosphatase to remove any exposed phosphate groups from the RV transcripts, or vaccinia capping enzyme to add a 5’ 7-methylguanine cap to any newly synthesized transcripts that had not been capped. Control reactions lacking these three enzymes were also set up. After the reactions were carried out, RNAs were purified and transfected into WT MEFs, and the level of secreted IFN-beta measured by ELISA 21 hr post-transfection. As shown in Figure 4A, we observed that 2’-O-methyltransferase-treatment did not decrease the IFN stimulatory potential of the *in vitro* ssRNA sample. However, treatment of the *in vitro* ssRNA sample with either antarctic phosphatase or capping enzyme significantly reduced its IFN stimulatory potential (Figure 4A). These results suggest that the *in vitro* ssRNA sample contains some uncapped mRNAs with exposed 5’ phosphates, but that the capped mRNAs that are produced appear to be fully methylated.

On the other hand, treatment of the *in vivo* 6 hr large RNA sample with any of the three enzymes significantly diminished its IFN stimulatory potential (Figure 4B). These results suggest that the *in vivo* 6 hr large RNA sample contains at least two distinct immunostimulatory RNAs: uncapped transcripts with exposed 5’ phosphate groups and capped transcripts that are incompletely 2’-O-methylated. In Figure 4C, the secreted IFN-beta concentrations from control polyI:C- and mock-transfected WT MEFs are shown.

## Discussion

In this report we provide new insight into how RV infection activates innate immune signaling. Although it has long been known that RV induces IFN signaling, and also efficiently blocks this pathway, the molecular basis for how RV triggers an innate response has remained unclear. Using an *in vitro*-transcription approach, we demonstrated that nascent RV ssRNA transcripts have IFN stimulatory potential, suggesting that they possess at least one PAMP. In a complementary approach, we isolated large RNA (>100 nt) fractions from cells infected with RV for 1 or 6 hours and determined that the 6 but not the 1 hour RNA sample also induced IFN signaling. Taken together, these results strongly suggest that RV RNA transcripts generated both *in vitro* with purified RV DLPs and *in vivo* in RV-infected cells contain immunostimulatory PAMPs. We further found that RIG-I is required for and MDA5 contributes to downstream innate immune signaling from these RNA samples. The RLR mitochondrial adaptor protein MAVS was also shown to be necessary for this response. Our findings confirm a recent postulation by Holloway and Coulson that RV plus-sense RNA transcripts could be possible RIG-I and/or MDA5 ligands because they are localized to the cytoplasm during RV infection and could potentially contain motifs known to trigger RIG-I/MDA5 signaling [57].

The results of enzymatic characterization assays carried out with the purified *in vitro* ssRNA and *in vivo* 6 hr large RNA samples indicate that some RV transcripts appear to be uncapped and contain exposed 5’ phosphate groups while other transcripts are capped but incompletely 2’-O-methylated. These data suggest that the VP3 component of the viral transcription machinery is not completely efficient at either adding a 5’ cap structure to nascent transcripts or adding a 2’-O-methyl group to capped transcripts, a proposition put forward in the literature [57,58]. As a result, RV infection produces RNA PAMPs that trigger an innate immune signaling cascade through the RLRs.

Until now, the only nucleic acid associated with *Reoviridae* viruses that has been shown to have immunostimulatory potential was genomic dsRNA [20]. It is possible that some newly synthesized RV genomic dsRNA could have been present in our *in vivo* 6 hr large RNA sample and could have been a source of IFN stimulation in our experiments. However, it is important to note that during RV infection, the dsRNA genome is generated during assembly of subviral particles. This process takes place in protein-rich viroplasm structures, which have been shown to protect viral RNAs from RNAi degradation [49]. As such, it seems unlikely that naked genomic dsRNA would be present in the cytoplasm of infected cells where the RLRs reside. Indeed, at 6 hpi we were unable to detect dsRNA in the cytoplasm of infected cells by immunostaining (Figure 2A).

Previous studies from our laboratory and others have determined that RV infection stimulates a MAVS-dependent early antiviral response that involves either RIG-I or MDA5, neither being absolutely required for signaling [45,46]. Our results are mostly consistent with these reports in that we found that MDA5 contributes to and MAVS is required for IFN production in response to both the *in vitro* ssRNA and *in vivo* 6 hr large RNA samples. However, in our experiments, we observed that RIG-I is indispensable for the IFN response to these RNA samples (Figure 3). One potential explanation for these different results is that our *in vivo* large RNA fraction was purified from cells at an early time-point post-infection, when the PAMPs that are produced may be primarily sensed by RIG-I. It is possible that later during infection, RV ligands that are primarily detected by MDA5, such as higher-order RNA structures or “webs”, may be generated as the level of RV transcription increases and RNAs accumulate in the cell [23].

In eukaryotes, newly transcribed RNAs initially contain a 5’ triphosphorylated end, but during subsequent processing of most cellular transcripts the exposed 5’ triphosphate ends are converted to a monophosphate or a guanine cap. The type I cap structure of many higher eukaryotic mRNAs is modified with a methyl group at the N7 position of the guanosine capping residue and the ribose-2’-O position of the 5’-penultimate residue (m^7^GpppGm). In addition to enhancing mRNA stability and translational efficiency, the 5’ cap structure is one way that host cells discriminate self from non-self RNA. The ability of viruses to activate RIG-I signaling through the production of uncapped RNAs with exposed 5’ phosphate groups is well-described in the literature [13,14,15]. Many viruses replicate in the cytoplasm and have therefore evolved their own capping and methylation machinery or mechanisms to “snatch” the caps from host mRNAs in order to mimic self-RNA and avoid detection by the host. N7 methylation of the 5’ cap is important for mRNA processes such as transcription elongation, polyadenylation, splicing, nuclear export, and efficient translation [59]. Until recently, the function of 2’-O-methylation of 5’ cap structures was unknown. A few years ago, Daffis and colleagues discovered that a West Nile virus mutant lacking 2’-O-methyltransferase activity was attenuated in wild type cells, but pathogenic in cells with a defective IFN signaling pathway. They found that cap 2’-O-methylation prevented sequestration by IFIT proteins, which act downstream of IFN production as regulators of protein translation [60]. Since then, Zust et al. has demonstrated that coronavirus 2’-O-methyltransferase mutants are more potent IFN inducers than wild type viruses and that MDA5 was required for the IFN response to viral mRNAs lacking 2’-O-methyl modification [24].

In our studies, we found that the immunostimulatory potential of the *in vitro* ssRNA sample was not sensitive to 2’-O-methyltransferase-treatment, which indicates that the VP3 methyltransferase activity in an IVT reaction is very efficient and that most of the capped transcripts are fully methylated. However, the immunostimulatory potential of the *in vivo* 6 hr large RNA sample was significantly reduced by 2’-O-methyltransferase-treatment (Figure 4). One possible explanation for the different results obtained with the *in vivo* and *in vitro* RNA samples is that in the IVT reaction the concentration of the methyl donor, SAM, may be higher than in an actual cell, and therefore the *in vitro* methylation reaction may be more efficient. Interestingly, early efforts to characterize the 5’ and 3’ ends of RV genomic dsRNA purified from DLPs determined that their 5’ cap structures are only partially 2’-O-methylated on the 5’-penultimate residue [56], consistent with the results of our *in vivo* experiments.

Although RV infection is clearly able to induce an innate immune response, there is abundant data demonstrating that RV can also effectively antagonize this pathway. RV has been shown to suppress IFN induction by targeting the transcription factors IRF3/5/7 for degradation through non-structural protein 1 (NSP1) [37,38,39,40,42,43]. In some strains, RV NSP1 was found to also target β-TrCP for degradation, thereby shutting off the NF-κB pathway, which is also required for IFN upregulation [41]. Recently, NSP1 was shown to interact with RIG-I and downregulate its protein levels, indicating another potential mechanism by which RV can antagonize the IFN response [61]. In other studies, RV has been shown to directly interfere with STAT1 activation [62]. NSP1 has been identified as an important genetic determinant of host range-restriction of murine RV replication in the murine intestinal tract [63,64], but other RV genes including VP3 and VP4 may also contribute to different replication phenotypes [65,66]. It is plausible that even minor variations in VP3 capping and methyltransferase activities could manifest as differential abilities to stimulate antiviral signaling, thereby limiting replication.

Our studies do not exclude the possibility that RV transcripts may contain other PAMPs that also activate IFN signaling through the RLRs. We carried out experiments with RNases that target base-paired regions of RNAs and our preliminary results indicate that RV transcripts contain structured regions (data not shown). This preliminary observation is supported by computer modeling and RNase mapping studies which suggest that RV plus-strand RNAs form panhandle structures through base-pairing between the 5’ and 3’ ends [67,68,69]. Such secondary and possibly tertiary structures within RV transcripts could constitute additional PAMPs for RIG-I and possibly MDA5. It is also possible that RV infection generates PAMPs that are sensed by other PRRs, such as TLR3, later in infection. Indeed *in vivo* studies in a mouse model of RV infection showed that age-dependent TLR3 expression within the intestinal epithelium may be implicated in susceptibility to RV infection [70]. Future work will be needed to determine if additional molecular patterns are produced at different time-points during RV infection. Increased understanding of the molecular basis for RV stimulation of innate immunity will be important in the design of more effective third generation live virus vaccines and targeted antiviral therapeutics.

## Materials and Methods

### Cells, viruses, and plasmids

MA104 African green monkey kidney cells were purchased from the American Type Culture Collection (ATCC) and were maintained in M199 media supplemented with 7.5% fetal calf serum (Invitrogen) and L-glutamine, penicillin, and streptomycin (complete M199). Huh7 and Huh7.5 human hepatoma cells [71,72] were a gift from Jeffrey S. Glenn (Stanford University School of Medicine) and were maintained in DMEM media supplemented with 10% fetal calf serum (Invitrogen) and L-glutamine, penicillin, and streptomycin (complete DMEM). Immortalized RIG-I wild type and knockout murine embryonic fibroblasts (MEFs) [73] were a gift from Michaela U. Gack (Harvard Medical School) and were maintained in complete DMEM media. Transformed MDA5 wild type and knock-out MEFs [74] were a gift from Marco Colonna (Washington University School of Medicine) and were maintained in complete DMEM media supplemented with non-essential amino acids (Invitrogen) and 1% beta-mercaptoethanol (Invitrogen) (enhanced DMEM). MAVS wild type and knock-out MEFs [75] were a gift from Zhijian Chen (University of Texas Southwestern Medical Center) and were also maintained in enhanced DMEM media. Simian RRV tissue culture-adapted rotavirus was propagated in MA104 cells in the presence of trypsin and titered by plaque assay as described previously [76]. pIFN-beta-luc (firefly luciferase) plasmid was a gift from Jae U. Jung (University of Southern California). pRL-TK (*Renilla* luciferase) plasmid was obtained from Promega.

### Preparation of rotavirus double-layer particles (DLPs)

Viral particles were first concentrated from RRV-infected MA104 cell lysates by genetron extraction and pelleting over a sucrose cushion. DLPs were then generated by treating the particles with 10 mM EDTA to remove the outer VP4/VP7 layer, followed by cesium chloride density gradient centrifugation as described [77,78]. DLPs were dialyzed against tris-buffered saline (TBS). DLP concentration was determined by Bradford assay. DLPs were aliquoted and stored at -80°C.

### *In vitro-transcriptions*

Complete *in vitro*-transcription (IVT) reactions were carried out using 2.4 μg of purified DLPs, 5 mM ATP, 2.5 mM GTP, 2.5 mM CTP, 2.5 mM UTP, 0.5 mM *S*-adenosylmethionine (SAM), 0.5 U/µl RNasin, and 1 mM DTT in 1X TNM buffer (35 mM Tris-HCl pH 8, 50 mM sodium acetate, and 7.5 mM magnesium acetate). Incomplete IVT reactions lacked ATP. Reactions were incubated for 5-6 hours in a 42°C water bath. Total RNA (genomic dsRNA and nascent transcripts) was purified by extraction with an equal volume of phenol: chloroform: isoamyl alcohol (P:C: IAA, Invitrogen) and precipitation with 0.3 M sodium acetate and ethanol. Total RNA was then sedimented by centrifugation, washed with 70% ethanol, dried briefly, and resuspended in RNase-free water. RNA concentration was determined by absorbance on a NanoDrop spectrophotometer (Thermo Scientific), and RNA samples were stored in aliquots at -80°C.

### Radiolabeling

A complete IVT reaction was carried out using 0.6 μg of purified DLPs, 5 mM ATP, 2.5 mM GTP, 2.5 mM CTP, 2.5 mM UTP, 1 mM α^32^P-GTP, 0.5 mM *S*-adenosylmethionine (SAM), 0.5 U/µl RNasin, and 1 mM DTT in 1X TNM buffer. An incomplete reaction lacking ATP and a reaction lacking DLPs were also carried out under identical conditions except for the indicated lacking constituent. Reactions were incubated for 5 hours in a 42°C water bath. Unincorporated radiolabel was removed by treating the IVT reactions with antarctic phosphatase (New England Biolabs) for 1.5 hours in a 37°C water bath. IVT samples were denatured in urea loading dye for 5 minutes at 95°C, cooled on ice for 5 minutes, and analyzed on a 20% urea-acrylamide gel (National Diagnostics) in 1X TBE buffer run at 500-700 V for approximately 4 hours until the bromophenol blue dye front migrated 15.5 cm. The gel was dried on Whatman paper overnight at 80°C, exposed to a phosphorimager screen overnight, and the screen was scanned using a Storm phosphorimager (Molecular Dynamics).

### Gel-purification of IVT RNAs

To isolate large and small molecular weight RNAs, IVTs were denatured with formamide for 5 min at 90°C and run on a denaturing 12% urea-acrylamide gel (National Diagnostics) in 1X tris borate EDTA (TBE) buffer (National Diagnostics) at constant 12 mA current for 4-6 hours until the bromophenol blue dye front was approximately 1 inch from the bottom of the gels. UV shadowing was used to visualize large and small molecular weight RNA bands, which were then excised from the gel and eluted in 0.5 M ammonium acetate, 10 mM magnesium acetate, 1 mM EDTA, and 0.1% SDS at 4°C overnight with nutation. RNA was separated from the gel material by centrifugation at full speed for 10 minutes, followed by extraction with an equal volume of P:C: IAA and precipitation with ethanol. RNA was then sedimented by centrifugation, washed with 70% ethanol, dried briefly, and resuspended in RNase-free water. RNA concentration was determined by absorbance on a NanoDrop spectrophotometer, and RNA samples were stored in aliquots at -80°C.

### Luciferase reporter assay

40,000 Huh7 human hepatoma cells were plated per well of a 24-well plate. After 24 hours, cells were transfected with 100 ng of pIFN-beta-luc (firefly luciferase) and 4 ng of pRL-TK (*Renilla* luciferase, Promega) using Lipofectamine 2000 (Invitrogen). After 24 hours, cells were mock-transfected, transfected with the entire recovered RNA sample, or transfected with 500 ng per well of polyI:C (Sigma) in duplicate using Lipofectamine 2000 (Invitrogen). Approximately 21 hours after RNA transfection, cells were lysed in 100 µl of passive lysis buffer (Promega), and an aliquot was analyzed using the dual-luciferase reporter assay system (Promega).

### Preparation of *in vitro* ssRNA sample

Isolation of the ssRNA fraction from the IVTs was carried out as described previously for bluetongue virus [55]. Briefly, DLPs were removed from the reaction mixture by tandem ultracentrifugation, followed by ssRNA precipitation using 2 M lithium chloride (LiCl). The ssRNA fraction was pelleted, resuspended in proteinase K buffer (10 mM Tris-HCl, 100 mM NaCl, 1 mM EDTA, and 0.5% SDS), and digested with 10 μg/ml of proteinase K for 30 minutes in 35°C water bath. ssRNA was deproteinized by extraction with an equal volume of P:C: IAA and an equal volume of chloroform: isoamyl alcohol (C: IAA, Fluka) and precipitated with 0.3 M sodium acetate and ethanol. ssRNA was then sedimented by centrifugation, washed with 70% ethanol, dried briefly, and resuspended in RNase-free water. RNA concentration was determined by absorbance on a NanoDrop spectrophotometer, and RNA samples were stored in aliquots at -80°C.

### Immunofluorescence

MA104 cells were seeded in 8-well chamber slides (Nunc) at 1.5 x 104 cells/well. After a 3-day incubation, cells were washed twice with M199 media supplemented with penicillin-streptomycin (incomplete M199) and either mock-infected, infected with RRV at an estimated MOI of 10, or transfected with 1 μg/well polyI:C (Sigma). After a one-hour incubation at 37°C, all the wells were aspirated, cells were washed once with incomplete M199, and incomplete M199 was added back to each well. After another 5 hours of incubation at 37°C, all wells were aspirated, cells were washed once with PBS, fixed for 10 minutes in 3% paraformaldehyde, washed once with PBS, and stored in PBS overnight at 4°C. J2 monoclonal antibody, which recognizes dsRNA with helix >40 basepairs, was purchased from English and Scientific Consulting, Hungary and used at a 1:500 dilution. Guinea pig anti-NSP5 polyclonal antibody was obtained from John Patton (National Institutes of Health) and was used at a 1:1000 dilution. Goat anti-mouse secondary antibody conjugated to Alexa 594 (Invitrogen) was used at a 1:250 dilution. Donkey anti-guinea pig secondary antibody conjugated to DyLight 549 (Jackson ImmunoResearch) was used at a 1:400 dilution in the presence of 1% donkey serum. All antibodies were diluted in immunofluorescence (IFA) buffer (3% bovine serum albumin, 1% saponin, 1% Triton X-100, 0.02% sodium azide). Primary antibody incubations were done at room temperature for approximately one hour and secondary antibody incubations were done at room temperature in the dark for approximately 45 minutes. After the primary antibody incubation, wells were washed three times with IFA buffer. After the secondary antibody incubation, wells were washed three times with IFA buffer and three times with PBS, chambers were removed from the slide, the slide was allowed to air-dry for a few minutes, coverslips were mounted with Vectashield mounting medium with 4',6-diamidino-2-phenylindole (DAPI) (Vector Laboratories), and the slides were sealed with nail polish and allowed to dry in the dark. Images were taken using a Zeiss LSM710 confocal microscope with the 40X objective and analyzed with Volocity 6.0.1 software (PerkinElmer).

### Purification of large RNA fraction from rotavirus-infected cells

T175 flasks of MA104 cells were infected with RRV at an MOI of 5 for 1 hour or 6 hours. Cells were washed three times with cold PBS, scraped into PBS, and pelleted 5 minutes at 1000 rpm. Cell pellets were resuspended in 3 ml of hypotonic buffer (10 mM HEPES pH 7.9, 1.5 mM MgCl2, 10 mM KCl, 1X protease cocktail inhibitor (Roche)), supplemented with 1 mM DTT, and incubated on ice for approximately 10 minutes to allow the cells to swell. Cells were then transferred to a Dounce homogenizer and broken with approximately 30 strokes to generate a cytoplasmic extract. The cytoplasmic extract was supplemented with 0.1% Triton X, and cell debris and nuclei were sedimented by centrifuging for 5 minutes at 1500 x g. The supernatant was digested with 100 μg/ml proteinase K in 1X SDS buffer (20 mM Tris-HCl pH 7.5, 0.5% SDS, 1 mM EDTA) for 20 minutes in a 42°C water bath. The total RNA sample was deproteinized by extraction with 2 volumes of P:C: IAA and 2 volumes of C: IAA and precipitated with 0.3 M sodium acetate and ethanol. Total RNA was sedimented by centrifugation, washed with 70% ethanol, dried briefly, and resuspended in RNase-free water. Total RNA was then digested with 2 U/µl DNase I (Ambion) for 30 minutes in 37°C water bath, supplemented with 1X SDS buffer and 0.3 M sodium acetate, extracted with 2 volumes of P:C: IAA and 2 volumes of C: IAA RNA, and precipitated with ethanol. Total RNA was then sedimented by centrifugation, washed with 70% ethanol, dried briefly, and resuspended in RNase-free water. RNA concentration was determined by absorbance on a NanoDrop spectrophotometer, and RNA samples were stored in aliquots at -80°C. To isolate large (>100 nt) RNAs from this total RNA fraction, total RNA was mixed with an equal volume of 5 M LiCl and incubated on ice for 30 minutes. The large RNA fraction was pelleted by centrifugation for 30 minutes at 14,000 rpm, resuspended in RNase-free water, and precipitated with 0.3 M sodium acetate and ethanol. Large RNAs were then sedimented by centrifugation, washed with 70% ethanol, dried briefly, and resuspended in RNase-free water. RNA concentration was determined by absorbance on a NanoDrop spectrophotometer, and RNA samples were stored in aliquots at -80°C.

### Enzymatic RNA characterization

*In vitro* ssRNA and *in vivo* 6 hr large RNA samples were denatured for 10 minutes at 65°C then quick-cooled on ice. 2’-O-methyltransferase (New England Biolabs), antarctic phosphatase (New England Biolabs), and 5’ capping (CellScript) reactions were set up using 10 μg of each RNA sample and the appropriate enzymes according to the manufacturer’s instructions. Control reactions lacking enzyme were also set up for each RNA sample. Reactions were incubated for 1.5 hours at 37°C followed by extraction with one volume of P:C: IAA and precipitated with 0.3 M sodium acetate and ethanol. RNAs were sedimented by centrifugation, washed with 70% ethanol, dried briefly, and resuspended in RNase-free water. RNA concentration was determined by absorbance on a NanoDrop spectrophotometer (Thermo Scientific) and RNA samples were stored in aliquots at -80°C.

**ELISA**. MEFs were seeded in 24-well plates at 4 x 10^4^ cells per well or 48-well plates at 2 x 10^4^ cells per well. When the wells reached 80-90% confluency, cells were mock-transfected or transfected with 500 ng per well (for 24-well plate) or 250 ng per well (for 48-well plate) of RNA or polyI:C (Sigma) using Lipofectamine 2000 (Invitrogen). After a 21-hour incubation, cell culture media was collected and the concentration of secreted IFN-beta protein in the cell media was measured using an enzyme-linked immunosorbent assay (ELISA) for mouse IFN-beta (PBL Biomedical Laboratories). The concentration of secreted IFN-beta was determined by comparison to a standard curve generated using a mouse IFN-beta standard according to the manufacturer’s instructions.
